# Supplementary material for: Phage parasites targeting phage homologous recombinases provide antiviral immunity
Source: Nat Commun. 2025 Feb 22;16:1889. doi: 10.1038/s41467-025-57156-3 (PMC11846896; doi:10.1038/s41467-025-57156-3)
Supplement: Supplementary file 1 — Supplementary Information [file 41467_2025_57156_MOESM1_ESM.pdf]

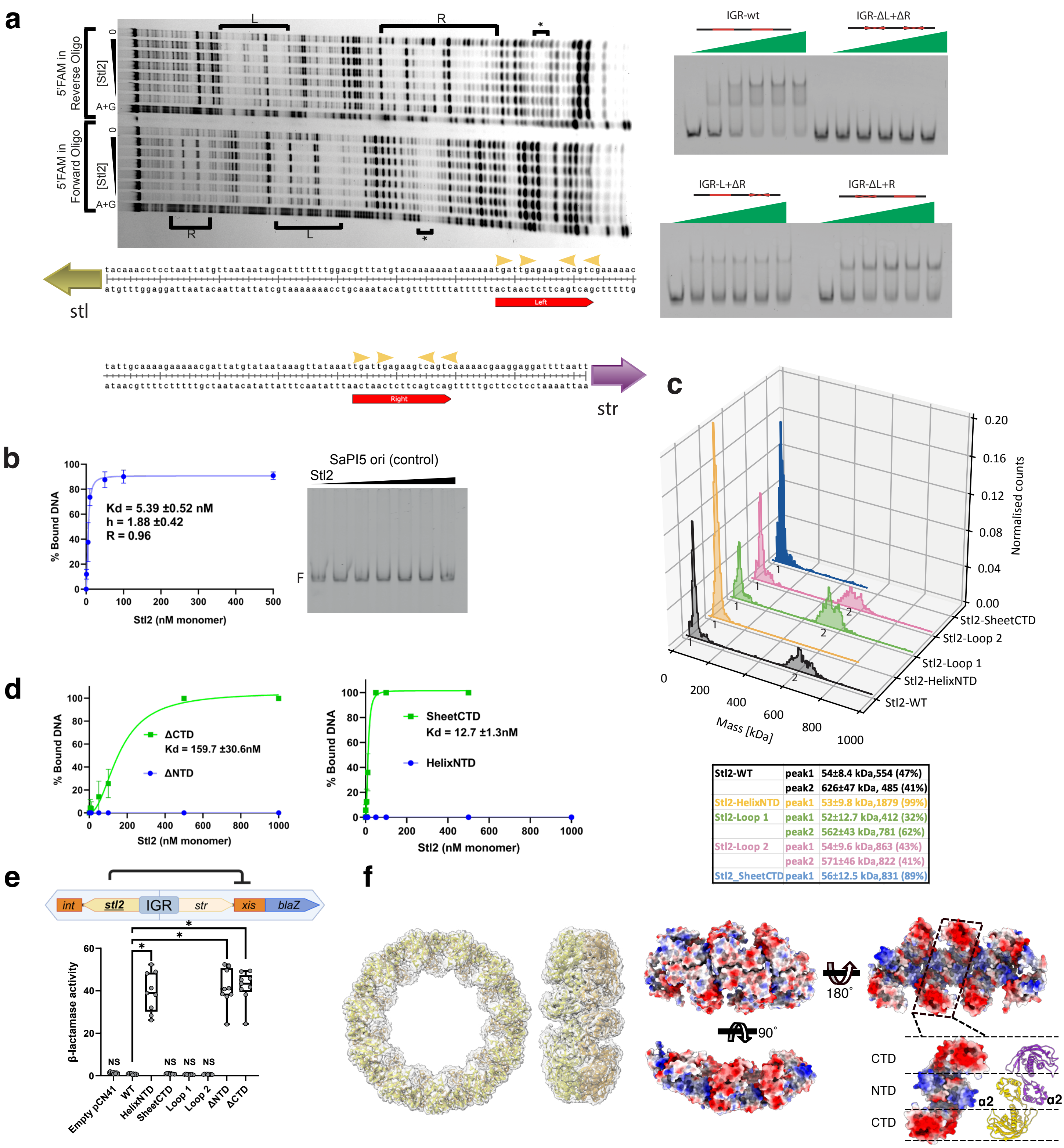

Stl2-WT	peak1	54±8.4 kDa,554 (47%)
	peak2	626±47 kDa, 485 (41%)
Stl2-HelixNTD	peak1	53±9.8 kDa,1879 (99%)
Stl2-Loop 1	peak1	52±12.7 kDa,412 (32%)
	peak2	562±43 kDa,781 (62%)
Stl2-Loop 2	peak1	54±9.6 kDa,863 (43%)
	peak2	571±46 kDa,822 (41%)
Stl2_SheetCTD	peak1	56±12.5 kDa,831 (89%)

Supplementary Figure 1. Related to Figure 2. **Characterization of Stl2**. **a**, top left, a representative DNAse I-based foot printing assay conducted on PCR-amplified IGR DNA with a 5'FAM label in either the forward or reverse oligos. Wells were loaded as follows: A+G ladder, 100nM, 500 nM, 1 μM, 2.5 μM, 5 μM, 10 μM and 0 nM of Stl2 (monomeric). Regions protected from DNAse I digestion are labelled as L (left), R (right) and marked with an asterisk. Bottom, sequence of the SaPI2 IGR, where the protected direct repeats are shown in red. The sequence TGATTGAGAAGTCAGTC(G/A) appears nowhere else throughout the SaPI2 genome. The yellow arrow heads on top mark the inverted sequences TGA, which were mutated in the EMSAs depicted on the right-hand side. The inverted TGA repeats were substituted to AAA in either left (IGR-ΔL+R), right (IGR-L+ΔR) or both (IGR-ΔL+ΔR) repeats. F, free DNA, B, bound DNA. **b**, quantification of the bands of at least the EMSA gels of wild-type Stl2 with SaPI2 IGR whose representative gel is shown in Figure 2A. The quantified bands were fitted with the Hill equation, where dots represent the mean values, and the error bars the standard deviations. Values shown are Kd (in nM), Hill coefficient (h) and R^2 (R). On the right-hand side, a SaPI5 PCR DNA at 2 nM was used as a negative control. F, free DNA. **c**, Corresponding mass distributions of the different Stl2 mutants analyzed by mass photometry as in Figure 2B. The mass (kDa), sigma (kDa), number and percentage of counts for each peak are shown below the graph. Since the counts measurements in the Stl2-sheetCTD mutant stopped at ~ 550 kDa, we further confirmed the oligomeric state of this mutant by SEC and cryo-EM visualization, both approaches confirmed that dimer was the only oligomeric state (not shown). **d**, the binding quantification of at least three gels whose representatives are shown in Figure 2E. The quantified bands were fitted with the Hill equation, where dots represent the mean values, and the error bars the standard deviations. Values shown are Kd (in nM). **e**, top, genetic segment of the SaPI2 genome fused to the β-lactamase gene present in the pCN41 shuttle vector. Stl2 transcriptionally represses the right-hand side operon of SaPI2. Bottom, nitrocefin-based β-lactamase *in vivo* assay of the RN4220 strains carrying pCN41 with the insert shown on top (WT) or the stl2 gene porting different mutations. β-lactamase activity is defined as increments in absorbance over time in minutes. See methods. Data are mean ± s.d, t-test, $p<0.0001$. NS, not significant respect the WT. **f**, Left, orthogonal views of Stl2's circles where the map is shown as transparent surface and the models of Stl2 dimers are shown as orange and yellow cartoons. This initial reconstruction was generated after imposing D11 symmetry and the map and model were not deposited in public databases, contrary to the three dimers reconstruction. Right, electrostatic surface representation of the Stl2 apo (three dimers). Top left, view of the convex surface, top right, view of the concave surface. The dashed box delimits one dimer, which is shown underneath along with the ribbon representation and colored as in Figure 2 D. The α-2 label marks the α-helix 2 that mediates both DNA binding and Stl2 oligomerization of dimers (Figure 2E, **c**, **d**). Individual replicates of all gels and graphs can be accessed in the source data provided as a Source Data file.

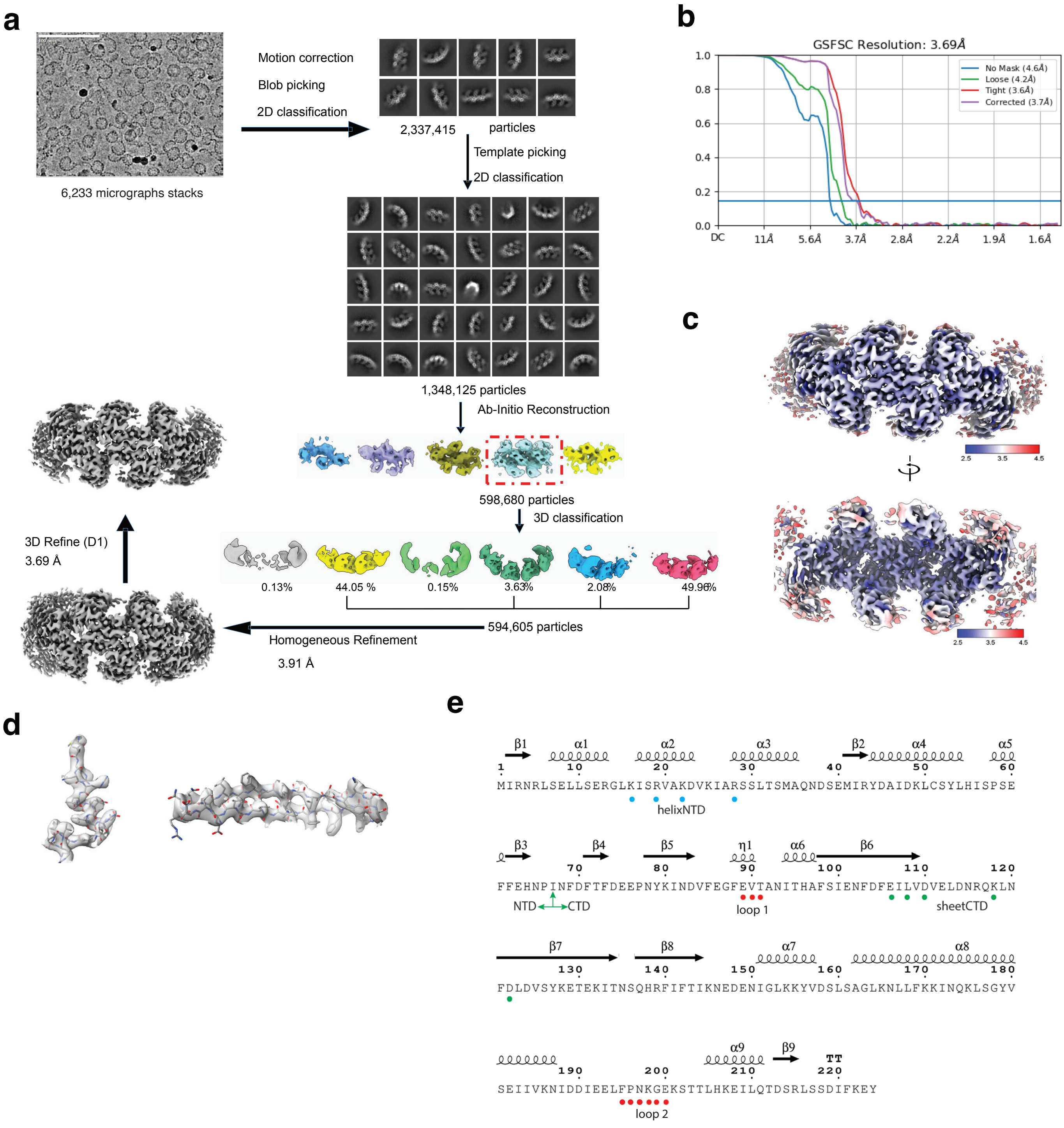

Supplementary Figure 2. **Stl2 cryo-EM reconstruction**. **a.** Pipeline for the 3D reconstruction. **b**. Global resolution estimation based on the gold-standard Fourier shell correlation (FSC). **c**. Final 3D reconstruction colored by local resolution, ranging from 2.5 Å to 4.5 Å. **d**. Representative regions of the map-model fit. **e**, Secondary structure elements are shown based on the structure of Stl2. The different mutations mentioned in the main text are highlighted as dots with the type of mutation labelled: blue dots mark mutations in HelixNTD, red dots in Loops 1 and 2, green dots mutations in SheetCTD. Green arrows mark the border (I67) between the NTD and the CTD.

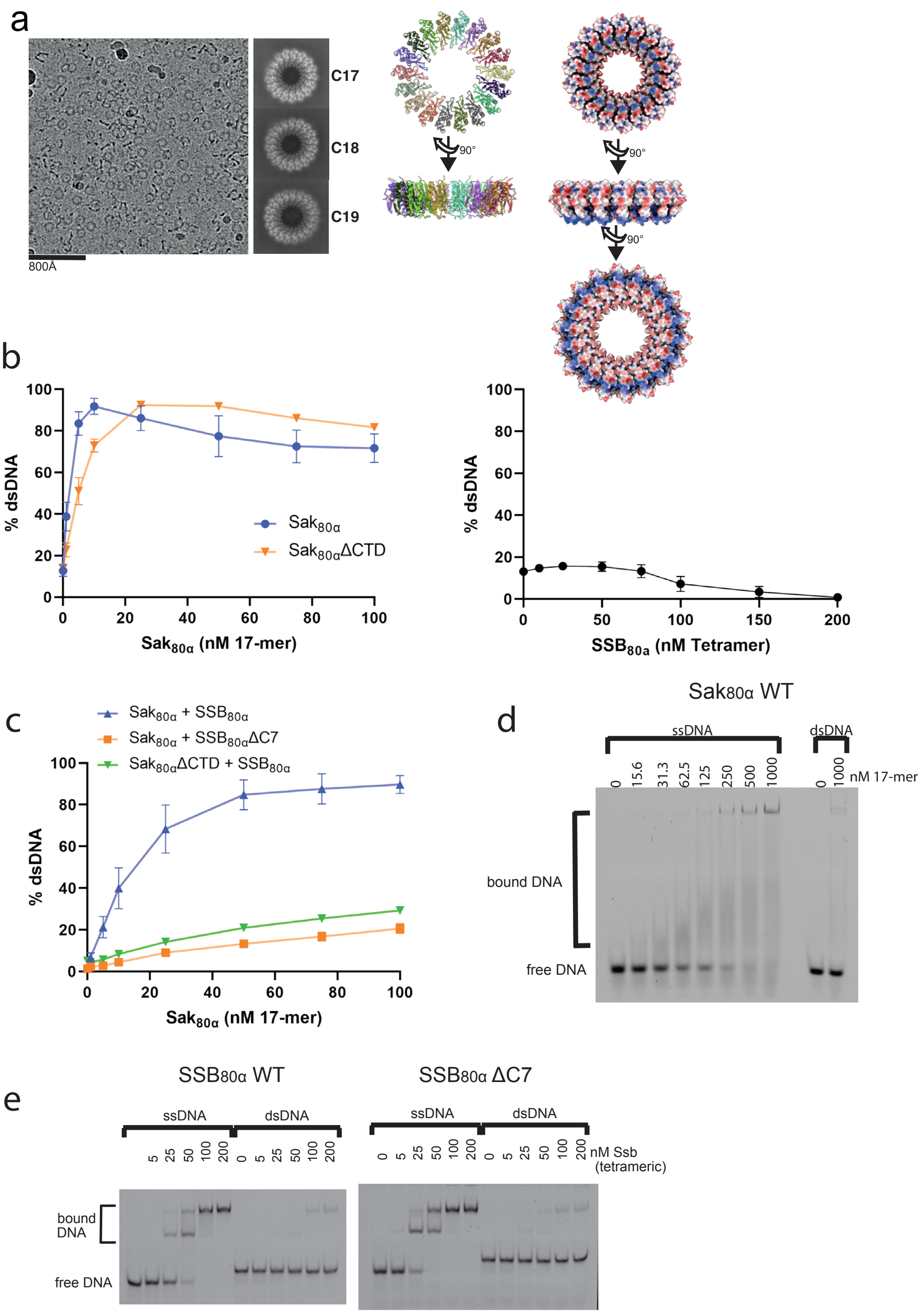

Supplementary Figure 3. **Cryo-EM reconstruction of $Sak_{80\alpha}\Delta CTD$ as an octadecameric torus and biochemical characterization**. **a,** Left, representative cryo-EM micrograph at 190000x magnification. Middle left, representative 2D classes showing $Sak_{80\alpha}\Delta CTD$ rings composed of 17, 18 or 19 protomers (top-, middle- and bottom-right, respectively). Middle right, orthogonal views of the model at 3.21Å after imposing C18 symmetry. The density in Figure 3A is colored according to each protomeric model here. Right, electrostatic surface representation of the model, rotated in increments of 90°. Red, electro negative, blue, electropositive. The blue patch in the periphery of the torus corresponds to the ssDBG **b**, left, curve graph quantifying dsDNA formation from at least three independent gels whose representatives are shown in Figure 3C. Values are represented as mean +/- standard deviations as error bars. Right, quantification of three $SSB_{80\alpha}$ annealing assays whose representative gel is shown in Figure 3D. **c**, quantification of at least three annealing reactions each shown in Figure 3E. **d**, EMSA where the stated concentration of $Sak_{80\alpha}$ was incubated with ssDNA (FAM-oligo-26) or dsDNA (FAM-oligo-26 annealed with oligo-25) at final DNA concentration of 2 nM. All gels were replicated three times. **e**, the stated concentrations of WT and ΔC7 $SSB_{80\alpha}$ were incubated with either ssDNA or dsDNA at the same final concentrations as in d. Individual replicates of all gels and graphs can be accessed in the source data provided as a Source Data file.

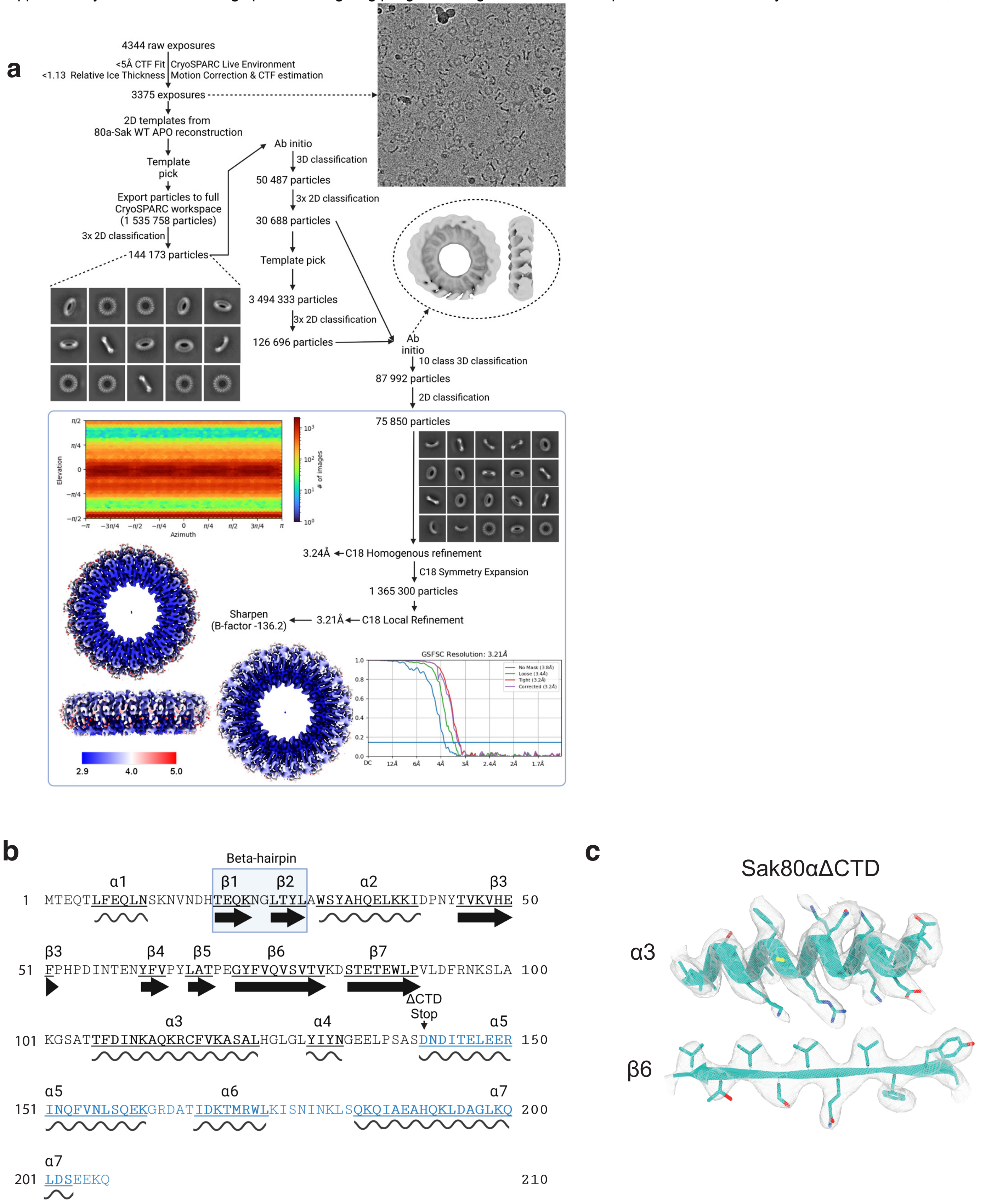

Supplementary Figure 4. **Structure determination of Sak80αΔCTD**. **a,** $Sak_{80\alpha}$ ΔCTD CryoEM data processing workflow. Local resolution of the finalized map is shown at the bottom together with the FSC curve and particle orientation distribution graphs from CryoSPARC. **b,** $Sak_{80\alpha}$ amino acid sequence and secondary structure. Residues comprising α-helices or β-strands are shown in bold and underlined and the secondary structure features are annotated (squiggly line for an α-helix, arrow pointing to the right for a β-strand). D141, highlighted with an arrow ("ΔCTD Stop"), is the residue at which the entire CTD is truncated in the ΔCTD mutant (all residues truncated in this mutant are shown in blue). **c**, Representative regions of the map-model fit. α-helix 3 and β-strand 6 are shown on the top and bottom respectively.

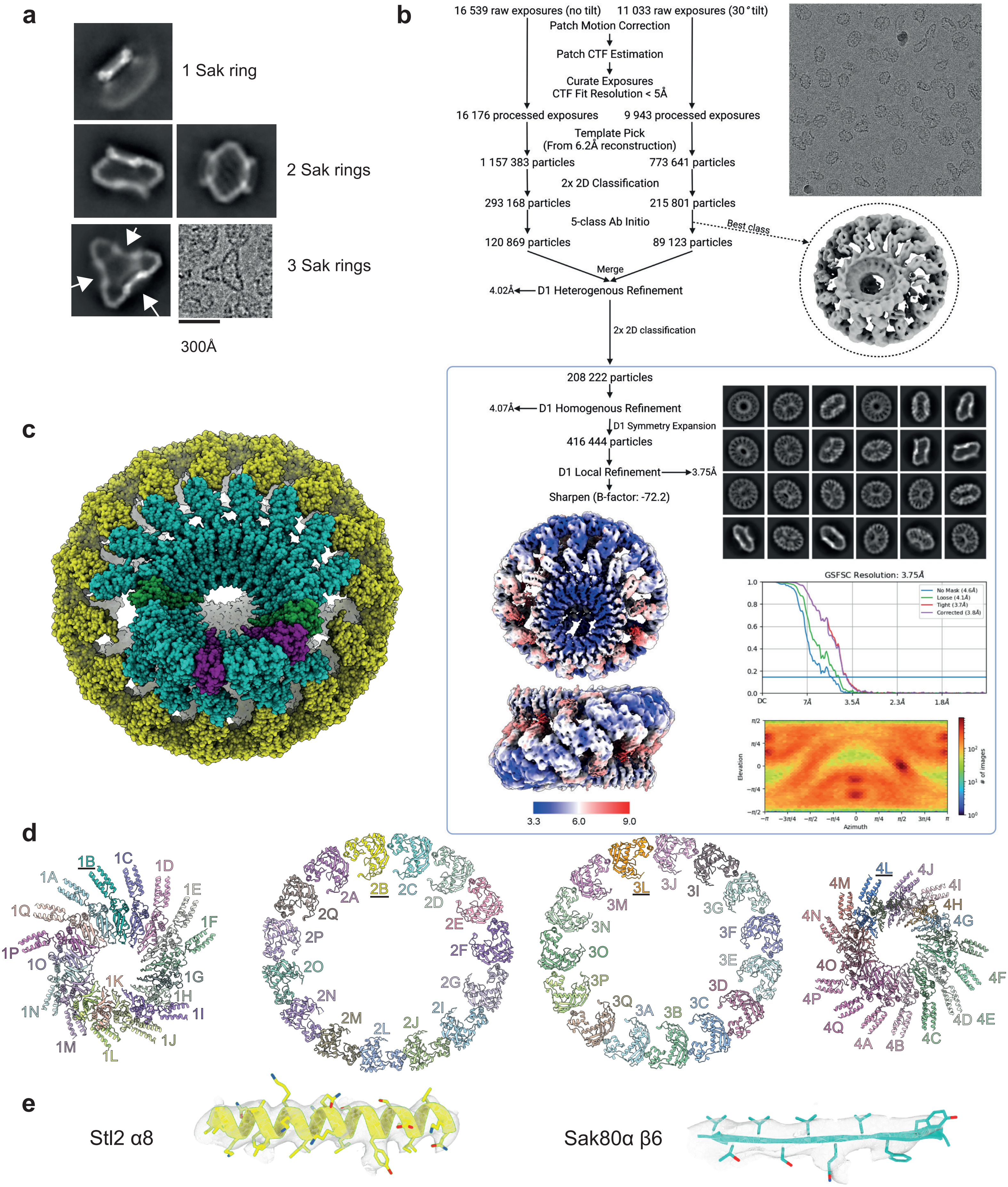

Supplementary Figure 5. **Stl2-Sak$_{80\alpha}$ data processing workflow**. **a,** Top, example of a 2D class showing only a single visible Sak$_{80\alpha}$ ring. Middle, example of two classes showing two Sak$_{80\alpha}$ rings connected by Stl2. Bottom left, an example of a 2D class where three Sak$_{80\alpha}$ rings can be seen connected by Stl2. Bottom right, cropped micrograph showing the triangular Sak$_{80\alpha}$-Stl2 variant. **b,** Cryo-EM data processing workflow. The local resolution map, FSC curve and particle distribution graph are shown at the bottom. **c,** Model with atoms shown as spheres. Sak$_{80\alpha}$ protomers with no CTD density are shown in purple. Sak$_{80\alpha}$ protomers with low quality CTD densities (which are not modelled) are shown in dark green. **d,** Chain identifiers used in the Sak$_{80\alpha}$-Stl2 model. Each of the rings in the overall model has been isolated for easier visualization. Chains 1A-1Q represent the Sak$_{80a}$ top ring (shown in Figure 5B), chains 2A-2Q represent one side of the dimeric Stl2 ring whose CTDs interact with the Sak$_{80a}$ top ring. In the same logic, chains 3A-3Q represent the second side of the Stl2 ring, interacting with the bottom Sak$_{80a}$ ring (chains 4A-4Q). The chains highlighted in Figure 5F are here underlined and shown in the same color whereas all other chains have been colored differently. **e,** Representative regions of the map-model fit. Stl2 αhelix 8 and Sak$_{80\alpha}$ β-strand 6 are shown on the left and right sides respectively.

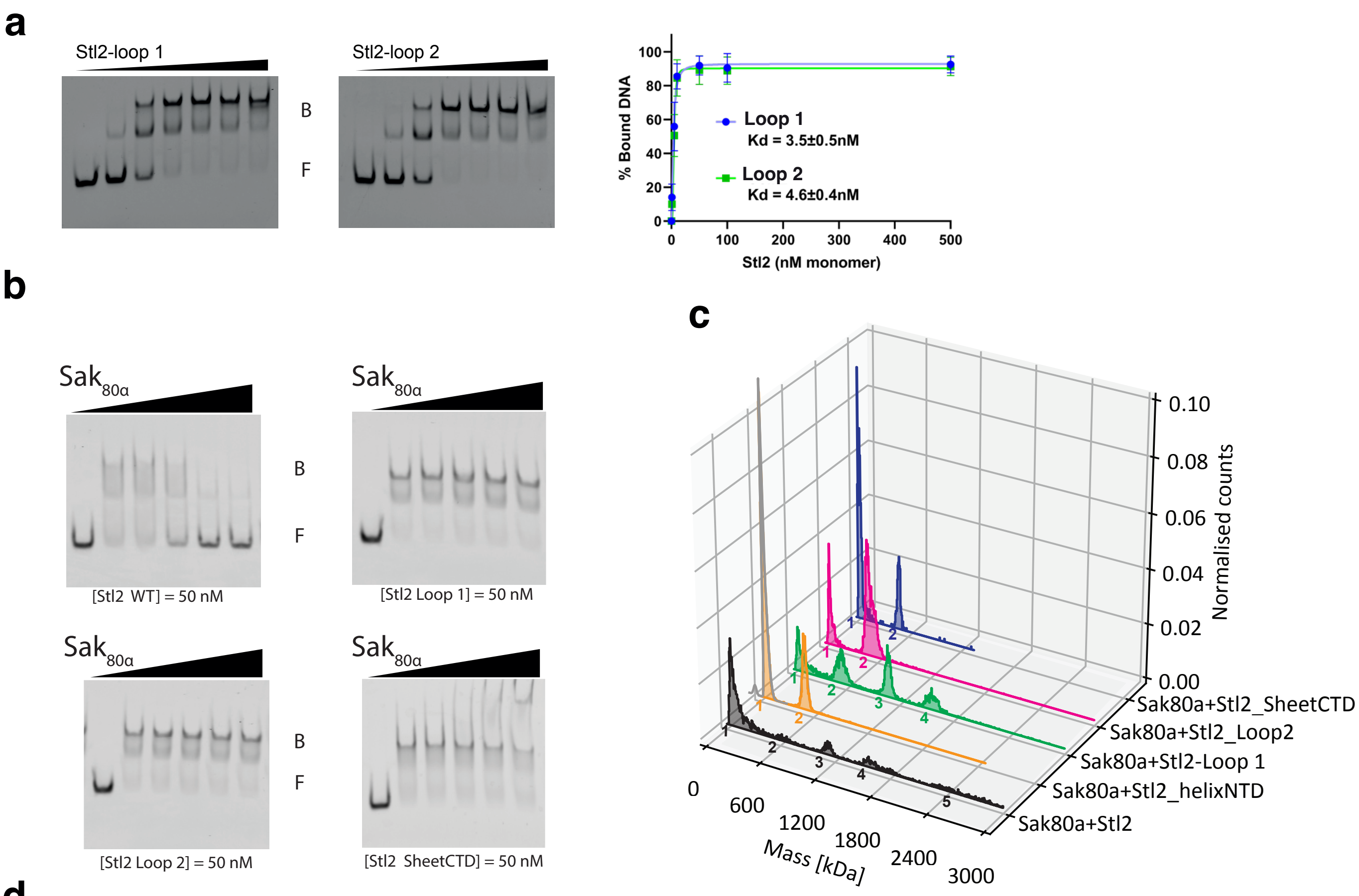

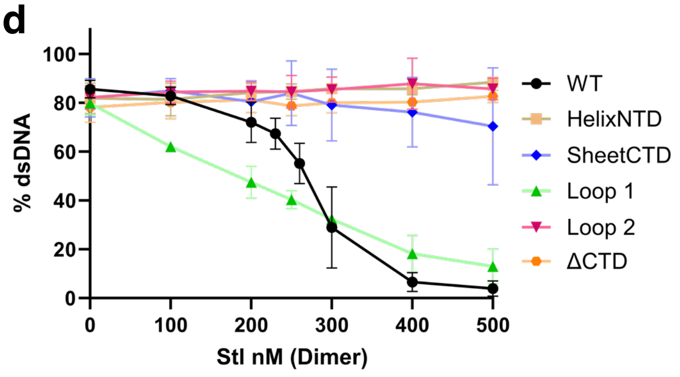

Sak80a-Stl2	peak1	161±98 kDa,2347 (47%)
	peak2	578±89 kDa,351 (7%)
	peak3	1129±50 kDa,408 (8%)
	peak4	1562±66 kDa,569 (11%)
	peak5	2529±253 kDa,621 (12%)
Sak80a+Stl2_helixNTD	peak1	66±21 kDa,5761 (70%)
	peak2	505±39 kDa,2380 (29%)
Sak80a+Stl2_Loop 1	peak1	59±14.8 kDa, 398 (10%)
	peak2	552±91 kDa,1150 (29%)
	peak3	1073±41 kDa,1044 (26%)
	peak4	1564±60 kDa,590 (15%)
Sak80a+Stl2_Loop2	peak1	62±28 kDa,1141 (24%)
	peak2	512±75 kDa,3563 (76%)
Sak80a+Stl2_SheetCTD	peak1	52±10.1 kDa, 416 (50%)
	peak2	503±16.4 kDa, 282 (34%)

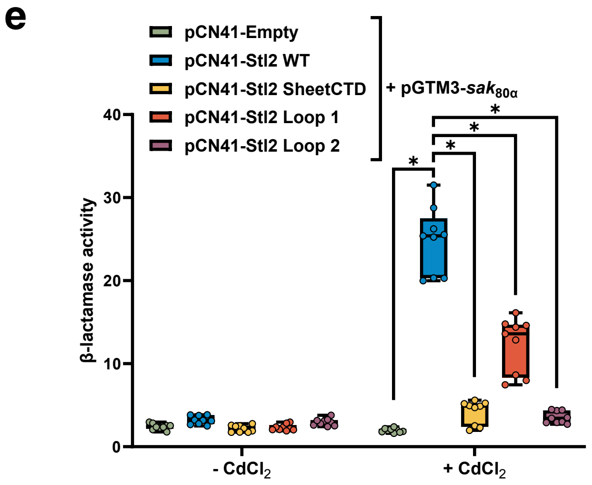

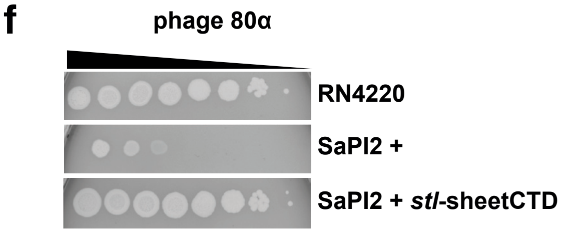

Supplementary Figure 6. **Structure-based analysis of the inhibition mechanism**. **a**, EMSAs showing the effect of the loop 1 and 2 mutants whose monomeric concentrations were the same as in Figure 2A. The graph on the right-hand side shows the binding quantification of at least three gels as in Supplementary Figure 1B. **b**, Same EMSAs as in Figure 4A with 50 nM Stl2 concentration instead of 100 of the variants Loop1, Loop2 and SheetCTD. **c**, Mass distribution of different versions of Stl2 in complex with Sak80α as per mass photometry. Mass (kDa), sigma (kDa), number and percentage of counts are depicted in the below table. **d**, Quantifications of the annealing assays as in Figure 4D employing different Stl2 mutants in the reaction mix. See text. **e**, Plasmid variants from Supplementary Figure 1E that did not demonstrate constitutive β-lactamase activity were electroporated into RN4220 carrying pGTM3 porting the *sak*$_{80\alpha}$ gene and tested for β-lactamase activity with and without addition of 5 μM $CdCl_2$ to induce *sak*$_{80\alpha}$ expression. β-lactamase activity is defined as increments in absorbance over time in minutes. See methods. Data are mean ± s.d, t-test, p<0.0001. **f**, Spot assay as in Figure 1, where ten-fold serial dilutions of phage 80α lysates were spotted on RN4220 strain, RN4220 harboring WT SaPI2 (SaPI2+) and RN4220 SaPI2+ carrying the *stl*-sheetCTD mutant variant. Individual replicates of all gels and graphs can be accessed in the source data provided as a Source Data file.

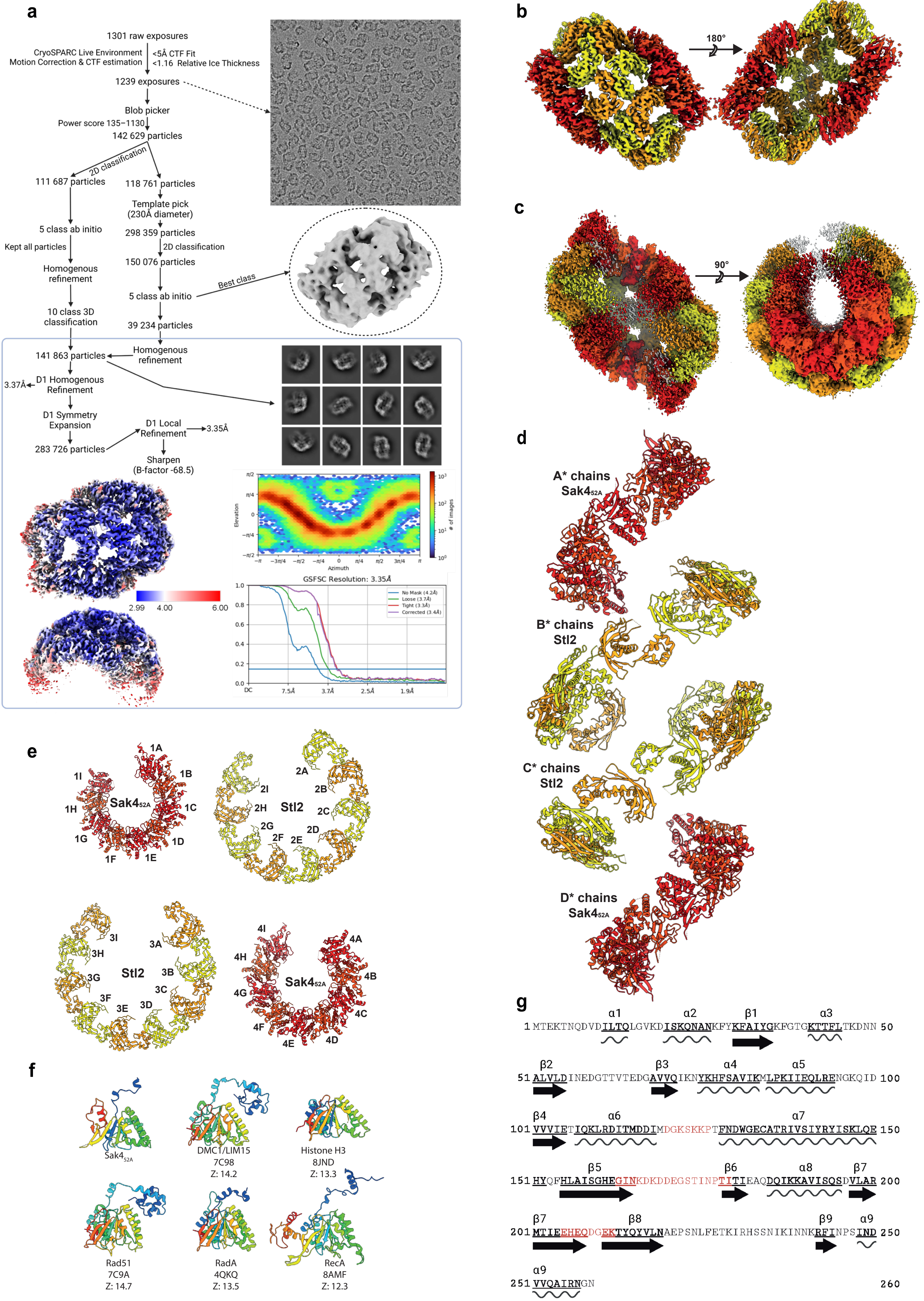

Supplementary Figure 7 legend in the next page

Supplementary Figure 7. **Sak4$_{52A}$-Stl2 cryoEM reconstruction and analysis. a,** Sak4$_{52A}$-Stl2 cryoEM data processing workflow. Local resolution is shown in the bottom left while the CryoSPARC FSC curve and particle orientation graphs are shown in the bottom right. **b.** The Sak4$_{52A}$-Stl2 map colored by the derived models. Regions where Sak4$_{52A}$ is present are colored in red/dark red and Stl2 with yellow/orange. **c.** The same map as in b but with a lower threshold showing fragmented density. **d.** Dissection of the full Sak4$_{52A}$-Stl2 model derived from **b**. Two antiparallel Sak4$_{52A}$ pseudo-filaments are held together by an oligomer of Stl2 dimers. The Sak4$_{52A}$ chains 1* interface with the Stl2 chains in 2* which dimerize with the Stl2 in chains 3* that in turn interface with the Sak4$_{52A}$ in chains 4*. **e.** The same representation as **d** except all sections are rotated 90°, showing them from the "top". Chain identifiers are shown next to their respective protomers. **f.** The Sak4$_{52A}$ model from the Sak4$_{52A}$-Stl2 complex was isolated and queried in the Dali server against the entire PDB database. Selected models are shown with a rainbow representation where the N-terminus is blue and the C-terminus is red. **g**, Secondary structure representation of Sak4$_{52A}$. Regions forming α-helices or β-strands are shown in bold and underlined and the secondary structure shown with either a squiggly line or arrow, respectively. Regions not modelled in the cryo-EM complex are shown in red.

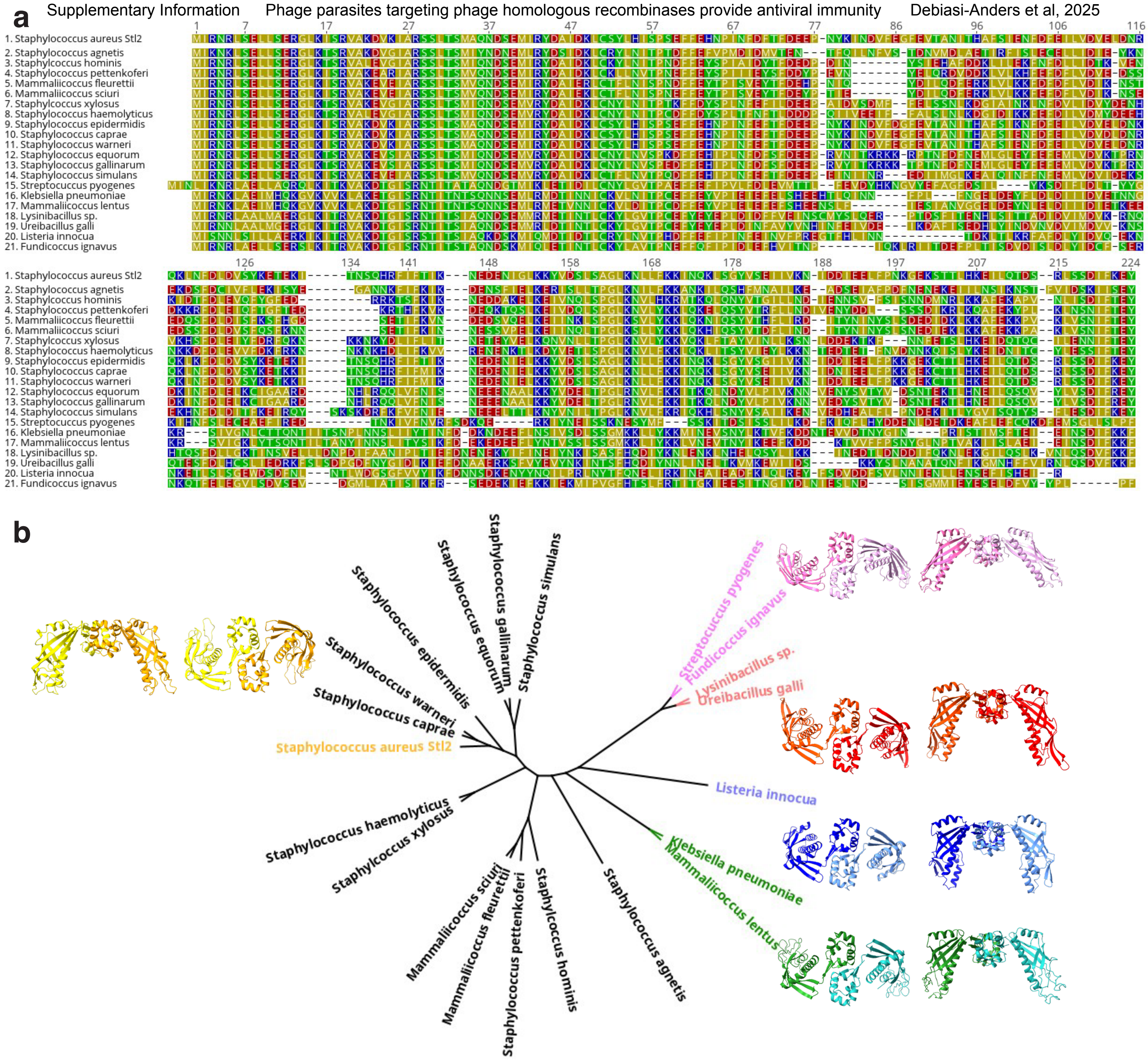

Supplementary Figure 8. **Stl2 Homologs intra and extra staphylococcal genus. a**. Multisequence MUSCLE alignment of 20 homologs retrieved by PBlast whose sequences showed similarities beyond the Stl2 NTD. The amino acid residues are colored by polarity as Geneious softwares settings. Stl2 sequence has been moved to the first row for clarity. The numbers above the sequence are the position within Stl2 sequence. **b**. Unrooted tree of the same 20 sequences as in A. Stl2 is colored yellow while the four most distant clusters are colored pink, red, blue and green. The sequence of Stl2 and one representative of each distant cluster (*Streptococcus pyogenes*, *Ureibacillus galli*, *Listeria innocua, Klebsiella pneumoniae*) have been folded using Alphafold2 Multimer algorithm and orthogonal views of the dimers are shown in the periphery to evidence the peculiar butterfly shape.

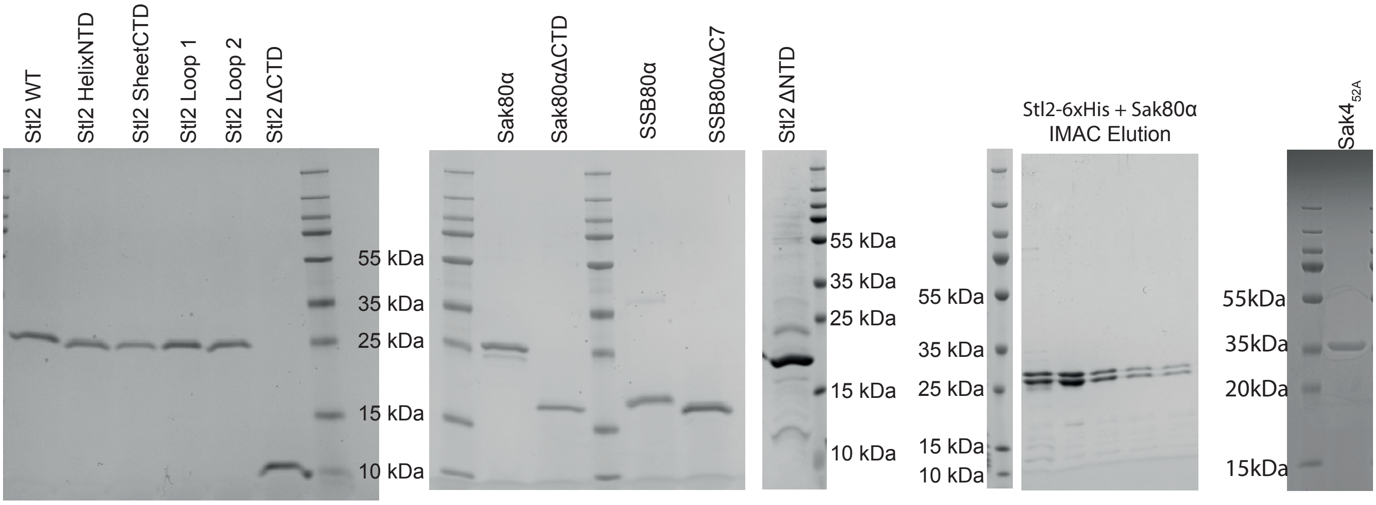

Supplementary Figure 9. **Purification of the proteins used in this study**. SDS Polyacrylamide Gel Electrophoresis of the different proteins and its variants.

Supplementary Table 1. Cryo-EM Data collection, model building and refinement statistics.

	Sak80αΔCTD-apo (EMD-17821) (PDB 8PQ8)	Sak80α-Stl2 (EMD-18346) (PDB 8QE9)	Stl2-apo (EMD-18248) (PDB 8Q86)	Sak4$_{52A}$-Stl2 (EMD-19048) (PDB 8RC5)
**Data collection and processing**				
Magnification	190,000	165,000	190,000	190,000
Voltage (kV)	200	300	200	200
Electron exposure (e–/Å^2)	50	50	50	50
Defocus range (μm)	-1.6 to -2.9	-1.0 to -2.0	-1.2 to -2.7	-1.2 to -2.4
Pixel size (Å)	0.746	0.704	0.746	0.746
Symmetry imposed	C18	D1	D1	D1
Initial particle images (no.)	3,494,333	1,931,024	2,337,415	142,629
Final particle images (no.)	75,850	208,222	594,605	141,863
Map resolution (Å) FSC threshold	0.143	0.143	0.143	0.143
Map resolution range (Å)	3.21	3.75	3.69	3.35
**Refinement**				
Initial model used (PDB code)	-	-	-	-
Model resolution (Å) FSC threshold	0.143	0.143	0.143	0.143
Model resolution range (Å)	3.21	3.75	3.69	3.35
Map sharpening *B* factor (Å^2)	-136.2	-72.2	-	-68.5
Model composition				
Non-hydrogen atoms	1052	104,484	9,171	64,360
Protein residues	128	12,818	1,109	7,866
Ligands	0	0	0	AGS: 16
B factors (min/max/mean)				
Protein	33.96/162.46/98.95	30/304.68/128.26	36.81/162.60/95.33	0/160.01/88.12
Nucleotide	-	-	-	-
Ligand	-	-	-	87.06/105.05/94.59
R.M.S. deviations				
Bond lengths (Å)	0.011 (0)	0.011 (0)	0.003 (0)	0.012 (0)
Bond angles (°)	1.504 (0)	1.469 (0)	0.570 (0)	1.539 (0)
Validation				
MolProbity score	0.88	1.36	1.65	1.18
Clash score	1.46	6.46	7.46	3.89
Poor rotamers (%)	0.00	0.45	0.29	0.25
Ramachandran plot				
Favored (%)	99.21	98.04	96.35	98.29
Allowed (%)	0.79	1.96	3.65	1.71
Disallowed (%)	0.00	0.00	0.00	0.00

Supplementary Table 2. Oligonucleotides used in this study.

ID	Oligo Name	Sequence (5'-3')	Description
1	FAM_Ori_SaPI5_F	[FAM]GAAAAGGTGAAGCTAAAAGCAATA	Control DNA for the EMSA.
2	Ori_SaPI5_HindIII_R	GGGAAGCTTTGAAATTTACAGCTATG CAACTCG	
3	1_SaPI2-Stl_FP_full-F-FAM	[FAM]GACAATCTATTTCTAATCAT	Generate PCR product of entire SaPI2 intergenic region. 5' FAM label on forward oligo.
4	2_SaPI2-Stl_FP_full-R	GTATTCGCTAAAATAGTCAT	
5	3_SaPI2-Stl_FP_full-F	GACAATCTATTTCTAATCATT	Generate PCR product of entire SaPI2 intergenic region. 5' FAM label on reverse oligo.
6	4_SaPI2-Stl_FP_full-R-FAM	[FAM]GTATTCGCTAAAATAGTCAT	
13	SakOrf16-F-NcoI	CATGCCATGGGGATGACTGAACAAACATTATT	Cloning 80a-*sak* to pET-His_1a, NcoI/BamHI restriction sites.
14	SakOrf16-R-BamHI	CGCGGATCCTTATTGTTTCTCCTCACTAT	
15	SsbOrf17-F-NdeI	GGAATTCCATATGTTAAATAGAACAGTATTAGT AGGA	Cloning 80a-*ssb* to pET-28a, NdeI/BamHI restriction sites.
16	SsbOrf17-R-BamHi	CGCGGATCCTCAGAACGGTAAGTCATCAT	
17	Stl_Duet_Ndei_F	GGGAATTCCATATGATTAGAAATAGATTGTC	Cloning SaPI2-*stl* to pET-21a, NdeI/XhoI restriction sites.
18	Stl_Duet_XhoI_R	CCGCTCGAGATATTCTTTAAAAATATCAC	
19	SaPI2-Stl-89-91-Ala_F	GGATTTGCAGCAGCAGCAAACATTACTCACGC TTTTTCG	Generate pIMS 1356, *stl2* Loop 1 mutant.
20	SaPI2-Stl-89-91-Ala_R	GCTGCAAACAAATCCCTCGAAAACATCATTAAT TTTA	
21	SaPI2-Stl-195-200-Ala_F	GATATTGAAGAGCTTGCAGCAGCAGCAGCAGC AAAAAGTACGACTCTACATAAAGAAA	Generate pIMS 1355, *stl2* Loop 2 mutant.
22	SaPI2-Stl-195-200-Ala_R	AAGCTCTTCAATATCGTCTATATTT	
23	SaPI2-Stl-SexMut_Ala_F	GTTGCAGCAGATGTAGCAATAGCAGCAAGTTC ACTTACTTCAATGGCAC	Generate pIMS 1178, *stl2* helixNTD oligomer mutant.
24	SaPI2-Stl-SexMut_Ala_R	TACATCTGCTGCAACTGCAGATATTGCTAGTC CTCTTTCTGACAGTAGTT	
25	SaPI2-Stl-Sheets-Ala-F	GAATTAGATAATAGGCAAGCATTAGCTTTTGCC TTAGACGTCTCATATAAAG	Generating pIMS 1378, *stl2* sheetCTD oligomer mutant.
26	SaPI2-Stl-Sheets-Ala-R	CCTATTATCTAATTCGACGGCTACTGCAATTGC AAAGTCAAAATTTTCAATC	
27	SaPI2-Stl-1-67-NTD-F	ATCCGATCCTCGAGCACCACCACCAC	Generating pIMS 1376, *stl2* ΔCTD (1-67).
28	SaPI2-Stl-1-67-NTD-R	GCTCGAGGATCGGATTATGTTCAAAAAATTCT GAAGGAG	
29	SaPI2-Stl-67-end-CTD-F	TACATATGATCAATTTTGACTTTACTTT	Generating pIMS 1377, *stl2* ΔNTD (67-225).
30	SaPI2-Stl-67-end-CTD-R	AATTGATCATATGTATATCTCCTTCTT	
30	80a-Sak-dCTD-F_v2	ATCCTTAGTCACTTGCACTTGGTAGTT	Generating pIMS 1218, $sak_{80α}$ΔCTD (1-141)
31	80a-Sak-dCTD-R_v2	CAAGTGACTAAGGATCCGAATTCGAGCT	
32	80a-SSB-C7-F	CTGCGATTTGAGGATCCGAATTCGAGCTCCG	Generating pIMS 1216, $ssb_{80α}$ΔC7, truncation of last 7 residues
33	80a-SSB-C7-R	GATCCTCAAATCGCAGTGGTATTATCAAAAGG ATTATTACC	
34	FAM-oligo-26	[FAM]TCCTTTTGATAAGAGGTCATTTTTGCGGA TGGCTTAGAGCTTAATTGC	Used in annealing assays, DNA binding (EMSA).
35	oligo-26	TCCTTTTGATAAGAGGTCATTTTTGCGGATGGC TTAGAGCTTAATTGC	
36	oligo-25	GCAATTAAGCTCTAAGCCATCCGCAAAAATGA CCTCTTATCAAAAGGA	
37	Stl_pET-MBP_NcoI_F	CATGCCATGGGCATTAGAAATAGATTGTC	Cloning to pETDuet-1: *stl2* to MCS #1 (with 6xHis tag,
38	SaPI2_Stl_6xHis_BamHI_R	CGCGGATCCTCAGTGGTGGTGGTGGTGGTGG CTGCCATATTCTTTAAAAATATCAC	

ID	Oligo Name	Sequence (5'-3')	Description
39	Sak_Duet_BglII_F_v2	GGAAGATCTCACTGAACAAACATTATTTGA	NcoI/BamHI) and untagged *sak*$_{80α}$ to MCS #2 (BglII/XhoI).
40	Sak_Duet_XhoI_R	CCGCTCGAGTTATTGTTTCTCCTCACTAT	
41	SaPI2-BamHI-F	CGCGGATCCATACGATAGCGCCATGTA	Cloning the *stl2-str2* region from SaPI2 to pCN41.
42	SaPI2-xis-KpnI-R	CGGGGTACCTGCCATTATTAATAAGTAATTCTTCA	
43	80a-Sak-FLAG-SalI-1F	GACTACAAAGACGATGACGACAAGACTGAACAA ACATTATTTGA	Cloning N-terminally FLAG-tagged *sak*$_{80α}$ to pGTM3. PCR #1: primers 43+45, PCR #2: primers 44+45. PCR #1 used as template for PCR #2, which becomes the FLAG-tagged insert.
44	80a-Sak-FLAG-SalI-2F	ACGCGTCGACCAGAAACTATTGAGTACGAGGAG GTAGAACATGGACTACAAAGACGATGA	
45	80a-Sak-BamHI-R	CGCGGATCCTTATTGTTTCTCCTCACTAT	
46	SaPI2-Stl-CTD-MiniS2-F	AAATTGATCATTACAAACCTCCTAATTA	Generating *stl2* ΔNTD (1-67) in pCN41.
47	SaPI2-Stl-CTD-MiniS2-R	TTGTAATGATCAATTTTGACTTTACTTT	
48	Stl2-dCTD-pCN41-F	CTCGATTATTATGTTCAAAAAATTCTGAAGGAG	Generating *stl2* ΔCTD (1-67) in pCN41.
49	Stl2-dCTD-pCN41-R	AACATAATTAATCGAGGTGATCAAATGG	
50	Phi52A-Sak4_NdeI_F_v2	GGGAATTCCATATGACTGAAAAAACTAATCAAGAT	Cloning *sak4*$_{52A}$ into pET-28a, NdeI/XhoI restriction sites.
51	Phi52A-Sak4_XhoI_R_v2	CCGCTCGAGTTAATTACCATTTCTAATTGCTTGTA	
52	Stl2-pMAD-BamHI-F	CGCGGATCCTAAAATAGTCATAATTAAAATCCTCCT TCG	Insertion of region 500bp up and down of sheetCTD mutation site into pMAD
53	Stl2-pMAD-EcoRI-R	CCGGAATTCCGGTGCGATATAATCGCC	
54	SaPIbov1-pCN41-BH-F	CGCGGATCCCGTCTTTCTTCTTATATTTTTTAATC	Produce *stl2-ppi2 and stl*bov1-*ppi*bov1 inserts (same *ppi* sequence, thus same reverse primer). Δ*stl*bov1 produced with IMS 104 as template instead of IMS 99.
55	SaPI2-pCN41-BH-F	CGCGGATCCCGTTTTTCATAACTTGCCAT	
56	SaPI2-dSTL-pCN41-BH-F	CGCGGATCCCAGACAATCTATTTCTAATCATTAC	
57	SaPI2/bov1-pCN41-pif-KI-R	CGGGGTACCTTGTTCACTTTTAACTAATTCAATC	
58	SaPI1-Stl-BamHI-F	CGCGGATCCTGACATTAATCTGTTTCATATAAAAT	Produce *stl*1-Δori1-*ppi*1 insert for pCN41
59	SaPI1-pCN41-full_pif-KI-R	CGGGGTACCTTAGACACACTCCGTTTC	
60	SaPI1-dOri-1R	TGTCCCACTACTGTCCTAAAAATAAGATGTTT	
61	SaPI1-dOri-2F	GGACAGTAGTGGGACACCTATCAA	
62	dStl1-F	CTCTTTTCATATAAATCACCTCTAATTATAATTAT	Mutation PCR to remove stl1 from pCN41 insert
63	dStl1-R	TTTATATGAAAAGAGACGCCTTATATAAA	

Supplementary Table 3. Plasmids used in this study.

	Vector	Cloned sequence	Enzymes	Forward ID	Reverse ID
pIMS 660	pET-His_1a	*sak*$_{80α}$	NcoI/BamHI	13	14
pIMS 1120	pET-21a	*stl2*	NdeI/XhoI	17	18
pIMS 1154	pETDuet1	*sak*$_{80α}$ and *stl2* (+C-terminal 6x-His)	NcoI/BamHI (*stl*) BglII/XhoI (*sak*)	37, 38	39, 40

	Vector	Cloned sequence	Enzymes	Forward ID	Reverse ID
pIMS 1356	pET-21a	*stl2* Loop 1 (E89, V90 and T91 to Ala)	NdeI/XhoI	19	20
pIMS 1355	pET-21a	*stl2* Loop 2 (F195, P196, N197, K198, G199, E200 to Ala)	NdeI/XhoI	21	22
pIMS 1178	pET-21a	*stl2*-helixNTD oligomer mutant (K16, R19, K22, R28 to Ala)	NdeI/XhoI	23	24
pIMS 1378	pET-21a	*stl2*-sheetCTD oligomer mutant (E106, L108, D110, K118, N120, D122 to Ala)	NdeI/XhoI	25	26
pIMS 1376	pET-21a	*stl2* ΔCTD (1-67)	NdeI/XhoI	27	28
pIMS 1377	pET-21a	*stl2* ΔNTD (67-225)	NdeI/XhoI	29	30
pIMS 1218	pET-His_1a	$sak_{80\alpha}$ΔCTD (1-141)	NcoI/BamHI	30	31
pIMS 662	pET-28a	$ssb_{80\alpha}$	NdeI/BamHI	15	16
pIMS 1205	pET-28a	$ssb_{80\alpha}$ΔC7, truncation of last 7 residues	NdeI/BamHI	32	33
pIMS 1404	pCN41	Empty vector	-	-	-
pIMS 448	pGTM3	Empty vector	-	-	-
pIMS 1473	pCN41	*stl2* to *str2*	BamHI/KpnI	41	42
pIMS 1487	pGTM3	FLAG-$sak_{80\alpha}$	SalI/BamHI	43/44	45
pIMS 1503	pCN41	*stl2* helixNTD to *str2*	BamHI/KpnI	23	24
pIMS 1505	pCN41	*stl2* sheetCTD to *str2*	BamHI/KpnI	25	26
pIMS 1565	pCN41	*stl2* Loop 1 to *str2*	BamHI/KpnI	19	20
pIMS 1509	pCN41	*stl2* Loop 2 to *str2*	BamHI/KpnI	21	22
pIMS 1510	pCN41	*stl2* ΔNTD to *str2*	BamHI/KpnI	46	47
pIMS 1594	pCN41	*stl2* ΔCTD to *str2*	BamHI/KpnI	48	49
pIMS 1340	pET-28a	$sak4_{52A}$	NdeI/XhoI	50	51
pIMS 1853	pMAD	Wild type *stl2* 500bp up/down	BamHI/EcoRI	52	53
pIMS 1856	pMAD	sheetCTD *stl2* 500bp up/down	BamHI/EcoRI	25	26
pIMS 1481	pCN41	*stl2-ppi2*	BamHI/KpnI	55	57
pIMS 1483	pCN41	Δ*stl2-ppi2*	BamHI/KpnI	56	57
pIMS 2022	pCN41	*stl*bov1-*ppi*bov1	BamHI/KpnI	54	57
pIMS 2024	pCN41	Δ*stl*bov1-*ppi*bov1	BamHI/KpnI	54	57
pIMS 2078	pCN41	*stl*1-Δori1-*ppi*1	BamHI/KpnI	58/59	60/61
pIMS 2089	pCN41	Δ*stl*1-Δori1-*ppi*1	BamHI/KpnI	62	63

Supplementary Table 4. Strains used in this study.

Escherichia coli strains

Strain		Plasmid #1	Cloned sequence #1
IMS 660	DH5α	pIMS 660	$sak_{80\alpha}$
IMS 695	BL21(DE3)	pIMS 660	$sak_{80\alpha}$
IMS 1120	DH5α	pIMS 1120	*stl2*
IMS 1123	BL21(DE3)	pIMS 1120	*stl2*
IMS 662	DH5α	pIMS 662	$ssb_{80\alpha}$
IMS 696	BL21(DE3)	pIMS 662	$ssb_{80\alpha}$
IMS 1154	DH5α	pIMS 1154	$sak_{80\alpha}$ and *stl2*
IMS 1155	BL21(DE3)	pIMS 1154	$sak_{80\alpha}$ and *stl2*
IMS 1356	DH5α	pIMS 1356	*stl2* Loop 1 (E89, V90 and T91 to Ala)
IMS 1360	BL21(DE3)	pIMS 1356	*stl2* Loop 1 (E89, V90 and T91 to Ala)
IMS 1355	DH5α	pIMS 1355	*stl2* Loop 2 mutant (F195, P196, N197, K198, G199, E200 to Ala)

Escherichia coli strains

Strain		Plasmid #1	Cloned sequence #1
IMS 1359	BL21(DE3)	pIMS 1355	*stl2* Loop 2 mutant (F195, P196, N197, K198, G199, E200 to Ala)
IMS 1178	DH5α	pIMS 1178	*stl2*-helixNTD mutant (K16, R19, K22, R28 to Ala)
IMS 1179	BL21(DE3)	pIMS 1178	stl2-helixNTD mutant (K16, R19, K22, R28 to Ala)
IMS 1385	DH5α	pIMS 1378	*stl2*-sheetCTD mutant (E106, L108, D110, K118, N120, D122 to Ala)
IMS 1378	BL21(DE3)	pIMS 1378	*stl2*-sheetCTD mutant (E106, L108, D110, K118, N120, D122 to Ala)
IMS 1383	DH5α	pIMS 1376	*stl2* ΔCTD (1-67)
IMS 1376	BL21(DE3)	pIMS 1376	*stl2* ΔCTD (1-67)
IMS 1384	DH5α	pIMS 1377	*stl2* ΔNTD (67-225)
IMS 1377	BL21(DE3)	pIMS 1377	*stl2* ΔNTD (67-225)
IMS 1218	DH5α	pIMS 1218	$sak_{80α}$ΔCTD (1-141)
IMS 1221	BL21(DE3)	pIMS 1218	$sak_{80α}$ΔCTD (1-141)
IMS 1205	DH5α	pIMS 1205	$ssb_{80α}$ΔC7, truncation of last 7 residues
IMS 1216	BL21(DE3)	pIMS 1205	$ssb_{80α}$ΔC7, truncation of last 7 residues
IMS 1404	DH5α	pIMS 1404	Empty pCN41
IMS 448	DH5α	pIMS 448	Empty pGTM3
IMS 1473	DH5α	pIMS 1473	*stl2* WT to *str2*
IMS 1487	DH5α	pIMS 1487	FLAG-$sak_{80α}$
IMS 1503	DH5α	pIMS 1503	*stl2* helixNTD to *str2*
IMS 1505	DH5α	pIMS 1505	*stl2* sheetCTD to *str2*
IMS 1565	DH5α	pIMS 1565	*stl2* Loop 1 to *str2*
IMS 1509	DH5α	pIMS 1509	*stl2* Loop 2 to *str2*
IMS 1510	DH5α	pIMS 1510	*stl2* ΔNTD to *str2*
IMS 1594	DH5α	pIMS 1594	*stl2* ΔCTD to *str2*
IMS 1340	DH5α	pIMS 1340	$sak4_{52A}$
IMS 1343	BL21(DE3)	pIMS 1340	$sak4_{52A}$
IMS 1853	DH5α	pIMS 1853	Wild type *stl2* 500bp up/down
IMS 1856	DH5α	pIMS 1856	sheetCTD *stl2* 500bp up/down
IMS 1481	DH5α	pIMS 1481	*stl2*-*ppi2*
IMS 1483	DH5α	pIMS 1483	Δ*stl2*-*ppi2*
IMS 2022	DH5α	pIMS 2022	*stl*bov1-*ppi*bov1
IMS 2024	DH5α	pIMS 2024	Δ*stl*bov1-*ppi*bov1
IMS 2078	DH5α	pIMS 2078	*stl*1-Δori1-*ppi*1
IMS 2089	DH5α	pIMS 2089	Δ*stl*1-Δori1-*ppi*1

Staphylococcus aureus strains

Strain		Plasmid #1	Cloned sequence #1	Plasmid #2	Cloned sequence #2
IMS 4	RN4220	-	-	-	-
IMS 63	RN4220	pIMS 1404	Empty pCN41	-	-
IMS 1494	RN4220	pIMS 1487	FLAG-$sak_{80α}$	-	-
IMS 1489	RN4220	pIMS 1473	*stl2* WT to *str2*	-	-
IMS 1602	RN4220	pIMS 1503	*stl2* helixNTD to *str2*	-	-
IMS 1603	RN4220	pIMS 1505	*stl2* sheetCTD to *str2*	-	-
IMS 1623	RN4220	pIMS 1565	*stl2* Loop 1 to *str2*	-	-
IMS 1604	RN4220	pIMS 1509	*stl2* Loop 2 to *str2*	-	-
IMS 1605	RN4220	pIMS 1510	*stl2* ΔNTD to *str2*	-	-
IMS 1606	RN4220	pIMS 1594	*stl2* ΔCTD to *str2*	-	-
IMS 1654	RN4220	pIMS 1487	FLAG-$sak_{80α}$	pIMS 1404	Empty pCN41
IMS 1496	RN4220	pIMS 1487	FLAG-$sak_{80α}$	pIMS 1473	*stl2* WT to *str2*
IMS 1523	RN4220	pIMS 1487	FLAG-$sak_{80α}$	pIMS 1505	*stl2* sheetCTD to *str2*
IMS 1631	RN4220	pIMS 1487	FLAG-$sak_{80α}$	pIMS 1565	*stl2* Loop 1 to *str2*
IMS 1525	RN4220	pIMS 1487	FLAG-$sak_{80α}$	pIMS 1509	*stl2* Loop 2 to *str2*
IMS 2016	RN4220	pIMS 1481	*stl2*-*ppi2*	-	-
IMS 2018	RN4220	pIMS 1483	Δ*stl2*-*ppi2*	-	-
IMS 2028	RN4220	pIMS 2022	*stl*bov1-*ppi*bov1	-	-
IMS 2029	RN4220	pIMS 2024	Δ*stl*bov1-*ppi*bov1	-	-
IMS 2083	RN4220	pIMS 2078	*stl*1-Δori1-*ppi*1	-	-
IMS 2090	RN4220	pIMS 2989	Δ*stl*1-Δori1-*ppi*1	-	-
IMS 1935	IMS105	pIMS 1487	FLAG-$sak_{80α}$	-	-
IMS 1936	IMS105	pIMS 448	Empty pGTM3	-	-

Strain		SaPI	Source/Reference
IMS 99	RN4220	SaPIbov1 *tst:tet*M	[1]
IMS 104	RN4220	SaPIbov1 *tst:tet*M *stl* mutant	[1]
IMS 105	RN4220	SaPI2 *tst:tet*M	*Provided by JR Penadés*
IMS 106	RN4220	SaPI1 *tst:tet*M	*Provided by JR Penadés*
IMS 108	RN4220	SaPI1 *tst:tet*M *stl* mutant	[1]
IMS 1872	RN4220	SaPI2 *tst:tet*M *stl*2::*stl*2-sheetCTD	*This work*

Supplementary References

1. Ubeda, C., Maiques, E., Barry, P., Matthews, A., Tormo, M.A., Lasa, I., Novick, R.P., and Penadés, J.R. (2008). SaPI mutations affecting replication and transfer and enabling autonomous replication in the absence of helper phage. Mol. Microbiol. *67*, 493–503. https://doi.org/10.1111/j.1365-2958.2007.06027.x.
